# Selection of reference genes for measuring the expression of *aiiO* in *Ochrobactrum quorumnocens* A44 using RT-qPCR

**DOI:** 10.1038/s41598-019-49474-6

**Published:** 2019-09-11

**Authors:** Dorota M. Krzyżanowska, Anna Supernat, Tomasz Maciąg, Marta Matuszewska, Sylwia Jafra

**Affiliations:** 1 Laboratory of Biological Plant Protection, Intercollegiate Faculty of Biotechnology, University of Gdansk and Medical University of Gdansk, University of Gdansk, A. Abrahama 58, 80-307 Gdansk, Poland; 2 Laboratory of Cell Biology, Intercollegiate Faculty of Biotechnology, University of Gdansk and Medical University of Gdansk, Medical University of Gdansk, Dębinki 1, 80-211 Gdansk, Poland

**Keywords:** Bacterial transcription, Bacterial genes

## Abstract

Reverse transcription quantitative PCR (RT-qPCR), a method of choice for quantification of gene expression changes, requires stably expressed reference genes for normalization of data. So far, no reference genes were established for the *Alphaproteobacteria* of the genus *Ochrobactrum*. Here, we determined reference genes for gene expression studies in *O. quorumnocens* A44. Strain A44 was cultured under 10 different conditions and the stability of expression of 11 candidate genes was evaluated using geNorm, NormFinder and BestKeeper. Most stably expressed genes were found to be *rho*, *gyrB* and *rpoD*. Our results can facilitate the choice of reference genes in the related *Ochrobactrum* strains. *O. quorumnocens* A44 is able to inactivate a broad spectrum of *N*-acyl homoserine lactones (AHLs) – the quorum sensing molecules of many Gram-negative bacteria. This activity is attributed to AiiO hydrolase, yet it remains unclear whether AHLs are the primary substrate of this enzyme. Using the established RT-qPCR setup, we found that the expression of the *aiiO* gene upon exposure to two AHLs, C6-HLS and 3OC12-HSL, does not change above the 1-fold significance threshold. The implications of this finding are discussed in the light of the role of quorum sensing-interfering enzymes in the host strains.

## Introduction

Reverse transcription quantitative PCR (RT-qPCR) is the most widely used approach for gene expression studies. However, despite being powerful, the method involves multiple steps and is sensitive to both flaws in experimental design and to technical variation.

One of the crucial decisions to make while performing a RT-qPCR experiment is the choice of stably expressed reference genes (RGs) for the normalization of data. In case of model or well-studied organisms, the selection of suitable RGs can be facilitated based on previous studies. For bacteria, literature provides a list of candidate RGs of potential general use due to their occurrence in multiple species. The most popular include *rho*, 23S rRNA, *rpoD*, *gyrB*^1^, *recA*^1–3^, 16S rRNA^4–6^, *dnaK*, *rpoB*^2,7^, *groEL*^7^, *gyrA*^8^. However, these candidates are not equally suitable for all species under all growth conditions and one should always experimentally verify the stability of a set of candidates when first-time applying RT-qPCR in the given (micro)organisms^9,10^. In 2009, a set of guideline was published called ‘Minimum Information for publication of Quantitative real-time PCR Experiments’ (MIQE)^11^. Its goal was to unify the quality of RT-qPCR results published in scientific journals, mainly concerning medical studies. In prokaryotic studies, with the exception of a few earlier works (i.a.^1^), the pursuit to meet the MIQE standards intensified only recently^10,12,13^. Still, in many publications concerning RT-qPCR in bacteria, the expression of the studied gene(s) is arbitrary normalized to certain RGs without validation of their expression stability or even without a clear explanation for the choice.

One of the bacterial genera for which no data has been available on the suitable RGs is *Ochrobactrum* spp. These ubiquitous *Alphaproteobacteria*, related to *Brucella*, *Agrobacterium* and *Rhizobium*^14–17^, attract scientific attention as opportunistic human pathogens^14,17^ but also as beneficial members of plant and nematode microflora and bioremediation agents^18–29^. In this study, we focused on the *Ochrobactrum quorumnocens* strain A44^30^, an isolate from the rhizosphere of potato formerly known as *Ochrobactrum* sp. A44^31^. As a result of the activity of AiiO hydrolase, A44 inactivates a broad spectrum of bacterial signaling molecules from the group of *N*-acyl homoserine lactones (AHLs)^32^. Many Gram-negative bacteria secrete and autodetect AHLs, responding at transcriptional level when a threshold concentration is reached. This regulatory mechanism, termed quorum sensing (QS), enables populations of single-celled organisms to perform coordinated actions such as bioluminescence, biofilm formation or production of virulence factors^33,34^. The functioning of QS can be disturbed by AHL-cleaving microorganisms like *O. quorumnocens* A44, resulting in the loss of the QS-dependent phenotype^35–37^. The latter phenomenon is known as the quorum quenching (QQ). For example, A44, when co-inoculated on plant tissue with the plant pathogenic bacterium *Pectobacterium parmentieri* SCC3193, attenuates the QS-governed virulence of this pathogen^31,38^.

To date, numerous AHL-inactivating enzymes have been described^39,40^. Discovery of many of them, including AiiO, resulted from screening studies designed for the selection of new QS-disrupting agents. It is unclear whether the cleavage of AHLs in some bacterial species is the primary function of the involved proteins^41^. For some of these enzymes, additional, unrelated functions were identified in the respective hosts, indicating that AHL cleavage may be the effect of catalytic promiscuity (reviewed in^40,41^). For example, BlcC (AttM) from *Agrobacterium tumefaciens* was found to be involved in the metabolism of gamma-aminobutyrate^42^, and PvdQ from *Pseudomonas aeruginosa* is necessary for the maturation of the pyoverdine siderophore^43^. Even if the function of certain enzymes is assumed to be inactivation of AHLs, the predicted biological purpose of this activity is not uniform among the QS-interfering strains. Depending on whether the strain produces its own AHL or not, the predicted functions include, but are not limited to, self-regulation of own QS, using AHLs as a source of nutrients and providing an advantage in the environment over the AHL-producing competitors (reviewed in^40^). The role of the AHL-cleaving enzyme AiiO in the metabolism and environmental fitness of *O. quorumnocens* A44 remains unclear.

In this study, we selected stably expressed RGs for RT-qPCR analyses in *O. quorumnocens* A44. Next, we applied RT-qPCR to measure changes in the expression levels of *aiiO* in A44 in response to AHLs and factors such as growth phase, growth temperature, the medium pH the addition of potato root extract. The study is a part of our investigation on the role of AiiO in the metabolism and fitness of *O. quorumnocens* A44.

## Results

### Selection of reference genes (RGs)

A *gfp*-tagged variant of *O. quorumnocens* A44 was used throughout the study (hereafter referred to as A44)^44^. Eleven candidate genes were explored as potential RGs. Nine candidate genes (16S rRNA, 23S rRNA, *dnaK*, *groEL*, *gyrB*, *recA*, *rho*, *rpoB* and *rpoD*) were chosen based on previous reports on RGs used in bacteria^1–7^ (Supplementary Table S1). The remaining two candidates were: *gfp*, constitutively expressed by the tagged strain from an artificially introduced vector pPROBE-GTkan^45^, and CES85_4722, an A44 gene encoding a hypothetical protein with no data concerning its stability (a blind control). The *gfp* was explored as an RG candidate due to its rare occurrence in the environment, a feature that would be valuable for potential studies on complex samples. Primers targeting the aforementioned genes in A44 were designed and specificity in amplification of fragments of expected sizes (139–148 bp) was confirmed by gel electrophoresis and a melt curve analysis (Supplementary Fig. S1AB). In parallel, the performance of the primers targeting the *aiiO* gene was verified.

### Expression stability of candidate RGs

A pilot assay was conducted to establish the expression stability of 11 candidate RGs under 10 different conditions (Table 1).

**Table 1:** Conditions for the growth of A44 strain applied for the RT-qPCR experiment.

Basal medium^a^	Supplements	pH^b^	Growth temp. (°C)	Growth phase^c^
LB (L)	—	5.5	28	early stat.
LB (L)4	—	7	28	early stat.
LB (L)	—	7	28	middle log.
LB agar (S)	—	7	28	O/N
LB agar (S)	—	7	20	O/N
LB agar (S)	—	7	37	O/N
LB agar (S)	co-culture with *P. parmentieri* SCC3193	7	28	O/N
M63 0.4% glucose (L)		7	28	O/N
M63 0.4% glucose (L)	50 μM C6-HSL^d^	7	28	O/N
M63 0.4% glucose (L)	50 μM 3OC12-HSL^e^	7	28	O/N
M63 0.4% glucose (L)	25% potato roots extract^f^	7	28	O/N

1(L) and (S) stand for liquid and solid medium, respectively.

^a^The medium was buffered only in case of pH 5.5. In other cases, 7 is the initial pH of the medium.

^b^If provided, growth phase was determined based on the growth curve of A44 GFP in LB at 28 °C. O/N – stands for an overnight culture (16–20 h). early stat. – stands for early stationary growth phase.

^c^Culture condition not included in the geNorm pilot experiment (initial ranking of RG candidates).

^d^C6-HSL – *N*-hexanoyl homoserine lactone.

^e^3OC12-HSL *– N*-3-oxododecanoyl homoserine lactone.

^f^The medium contained water extract from the roots of soil-grown, 3-week-old potato plants (final concentration 25%).

**Figure 1:**
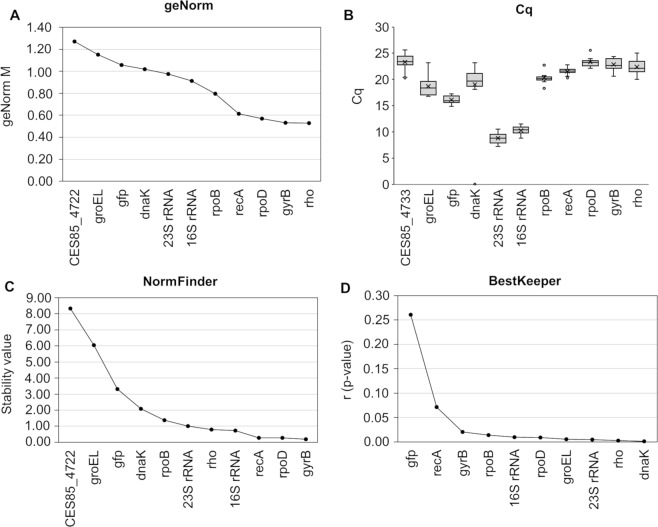
Expression stability of candidate RGs in *O. quorumnocens* A44 obtained using the respective algorithms: geNorm (**A**), NormFinder (**C**) and BestKeeper (**D**). Panel B shows Cq values for the investigated genes. The stability of expression of the RGs candidates was investigated under 10 different culture conditions. As BestKeeper enables simultaneous analysis of up to 10 genes, the blind control CES85_4722 (the eleventh gene), being the least stable gene according to geNorm and NormFinder, was excluded from the set for this tool.

According to geNorm^46^, the most stably expressed candidate RGs, characterized by the lowest geNorm M values (≤0.6), were found to be *rho, gyrB* and *rpoD*, followed by *recA* (Fig. 1A). The commonly applied RG encoding 16S rRNA was considerably less stable (M = 0.913). The heterologously expressed *gfp* gene, with M = 1.058, was also not among the top-ranking candidates. The mean threshold quantification cycle (C_q_) obtained for the 16S rRNA was 10.26 and 8.79 for the 23S rRNA. Therefore, the C_q_ for these RG candidates was 6–13 and 7–15 cycles lower, respectively, than that of the other candidates, reflecting the high abundance of the rRNA species in the cells (Fig. 1B).

The results obtained with geNorm were compared with RG ranks obtained with two other popular algorithms: NormFinder^47^ and BestKeeper^48^. The 4 genes selected by geNorm were also among the top 5 ranked by NormFinder, accompanied by the 16S rRNA gene (Fig. 1C). For three best-ranking genes, *gyrB*, *rpoD* and *recA*, NormFinder assigned stability values of 0.19, 0.28 and 0.28, respectively. BestKeeper ranked all analyzed genes except for *gfp* and *recA* as well-performing, that is with a statistically significant (α = 0.05) correlation between the correlation coefficients of individual genes with the BestKeeper index calculated for all genes in the analysis (Fig. 1D). A juxtaposition of the results from the three algorithms ranked *rho, rpoD* and *gyrB* as the most stable genes (Supplementary Table S2).

**Figure 2:**
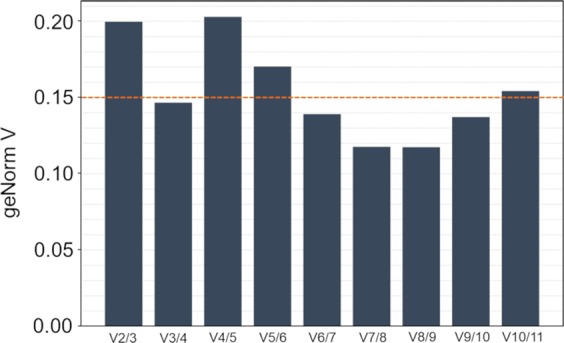
Optimal number of reference genes for normalization of RT-qPCR data suggested by geNorm. The tool calculates the pair-wise variation V_n_/V_n + 1_, where n is the number of reference genes used for normalization. According to Vandesompele and co-workers^46^, V value below 0.15 (dotted line) signifies that inclusion of an additional gene (n + 1) to the normalization factor yields no benefit.

Apart from ranking genes by their stability, the geNorm tool can also suggest the optimal number of RGs to be included in the normalization factor (NF)^46^. In this study, geNorm tool suggested using three RGs (V value < 0.15 threshold) (Fig. 2). Therefore, the complete set of samples (11 conditions, 2–3 biological replicates) for the analysis of the expression of *aiiO* (gene of interest) was analyzed by targeting *aiiO* alongside the three most stable RG candidates: *rho, rpoD* and *gyrB*. Next, the stability of expression of the applied RGs was validated on the complete dataset. geNorm analysis indicated the highest stability for *rho* (M = 0.441, CV = 0.187), followed by *gyrB* (M = 0.453, CV = 0.185) and *rpoD* (M = 0.5, CV = 0.221).

### PCR efficiency

PCR efficiency is a parameter included in the final calculation of the relative abundance of the investigated targets. With the primers designed in this study, PCR efficiency for *gyrB* and *rho* were 95.9% and 93.5%, respectively, and similar to the efficiency for the gene of interest *aiiO* (94.9%). The efficiency of PCR for *rpoD* was slightly lower (89%) (Table 2; Supplementary Fig. S2).

**Table 2:** Validation of RT-qPCR assay parameters for primers targeting *aiiO*, *gyrB*, *rho* and *rpoD*.

Parameter^a^	*aiiO* ^b^	*gyrB*	*rho*	*rpoD*
Efficiency (%)	94.9	95.9	93.5	89
R2	0.999	1	1	0.999
Slope	−3.451	−3.425	−3.487	−3.618

^a^Efficiency – calculated primer efficiency; R2 – correlation between log C_q_ and C_q_ in the standard curve; Slope – slope of the standard curve.

^b^Primers used for PCR amplification*: aiiO*: F_aiiO_OP7/R_aiiO_OP7; *gyrB*: F_ gyrB_OP7/R_ gyrB_OP7; *rho*: F_ rho_OP7/R_ rho_OP7 (Table S1).

### Comparison of the performance of the selected normalization factors (NFs)

**Figure 3:**
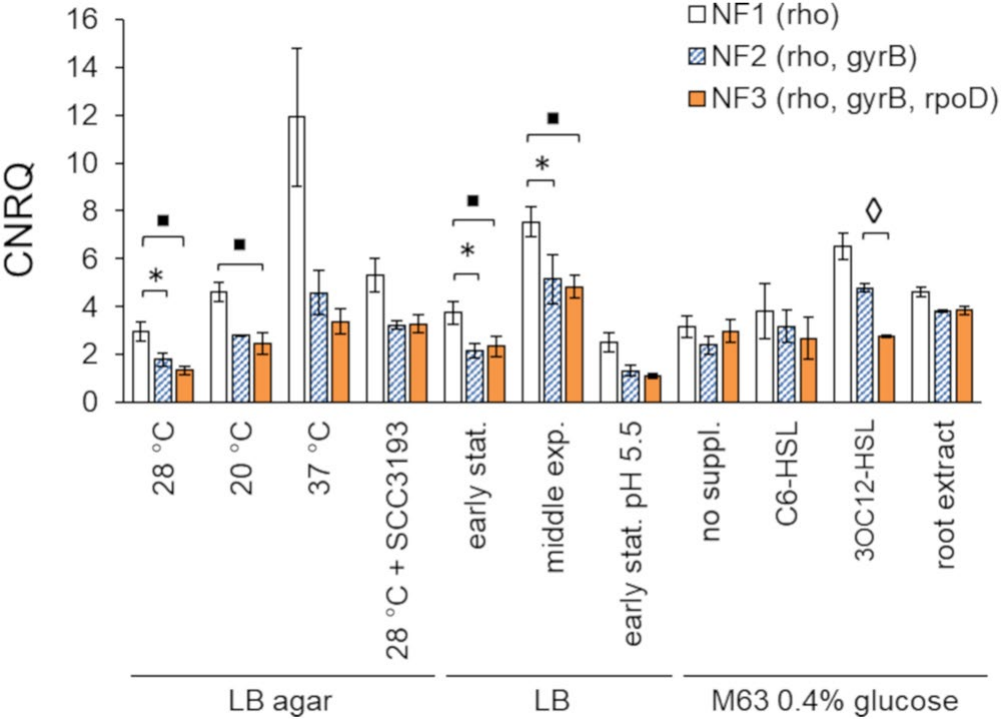
Influence of the type of the applied normalization factor (NF) on the relative expression of *aiiO* under different culture conditions. Statistically significant differences between groups (p < 0.05) are marked with: asterisk (NF1 vs NF2), squares (NF1 vs NF3) and diamonds (NF2 vs NF3). CNRQ – calibrated normalized relative quantity.

GeNorm analysis recommended applying 3 RGs for data normalization in the developed RT-qPCR assay. To determine whether the change of RGs included in the NF may influence the interpretation of the expression data for our gene of interest (*aiiO*), we compared the consistency of results obtained using 3 different NFs: NF1 (*rho*, the most stably expressed RG based on geNorm), NF2 (*rho*, *gyrB*) and NF3 (*rho*, *gyrB* and *rpoD*). For the comparison of the single-gene NF1 and the 2-gene NF2, the results were significantly different (p < 0.05) under 3 out of 11 culture conditions tested (LB agar 28 °C, LB early stationary, LB middle exponential) (Fig. 3). For the comparison of the single-gene NF1 and the 3-gene NF3, the results were significantly different in 4 conditions (LB agar 28 °C, LB agar 20 °C, LB early stationary, LB middle exponential). On the contrary, the difference between applying the 2-RG NF2 and the 3-gene NF3 was significant only under a single culture condition (M63 + 3OC12-HSL) (Fig. 3).

### Changes in the expression level of *aiiO* in response to AHLs and other culture conditions

Expression of *aiiO* was investigated in three types of media: lysogeny broth (LB) with peptone as the main energy source, LB solidified with agar and a defined mineral medium M63 supplemented with 0.4% glucose. Based on these media, a set of 11 conditions was established, 10 of which were included in the study on the stability of RGs (Table 1), in order to test whether the expression of *aiiO* in A44 is affected by factors such as: (i) the growth temperature, (ii) growth phase, (iii) addition of potato root extract and finally (iv) the presence of chosen AHLs. Two of the AHL types, *N*-3-oxo-octanoyl homoserine lactone (3OC8-HSL) and *N*-3-oxohexanoyl homoserine lactone (3OC6-HSL), were delivered as compounds secreted by the co-cultured cells of *P. parmentieri* SCC3193^38^. *N*-hexanoyl homoserine lactone (C6-HSL) and *N*-3-oxododecanoyl homoserine lactone (3OC12-HSL) were delivered as commercially synthesized pure compounds in 50 µM concentration. All AHLs included in the assay are known to be degraded by A44. To prevent complete depletion of AHLs prior sampling of cells for RNA extraction, the sampling was performed 90 min post addition of AHLs. The time point was chosen based on a previous experiment in which C6-HSL, supplemented to the culture in a 5-time lower concentration (10 µM), could still be detected in the supernatant of culture of A44 at 40 min (strong signal) and 100 min (weak signal) post addition^31^.

**Figure 4:**
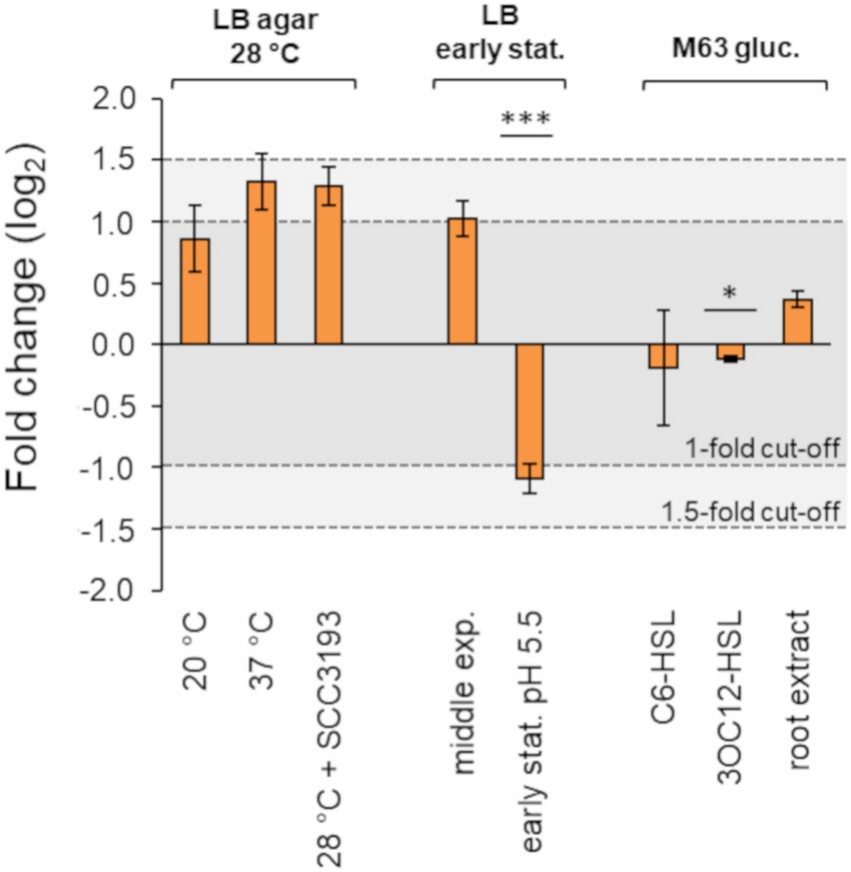
Fold change in relative expression of *aiiO* under different culture conditions. Relative expression (CNRQ) of *aiiO* in all samples was calculated with respect to normalization NF3 comprising *rho*, *gyrB* and *rpoD*. The fold change (log_2_) was calculated independently for each type of basal medium (LB agar, LB and M63 0.4% glucose) where the median CNRQ of one of the samples was treated as a reference (‘control’). If not indicated otherwise, the cells were grown at 28 °C. SCC3193 – growth in the presence of AHLs secreted by *P. parmentieri* SCC3193; no suppl. – without supplementation; early stat. – early stationary growth phase; middle exp. – middle exponential growth phase; C6-HSL, 3OC12-HSL – *aiiO* expression 90′ after the addition of 50 μM·mL^−1^ of the respective AHL; root extract – growth in the presence of 25% water-based extract obtained from potato roots. *denotes p < 0.05 ***denotes p < 0.0005 calculated using BootstRatio.

The expression data for *aiiO* was normalized with respect to NF3 and the resulting CNRQs (Supplementary Table S3) were grouped by the type of basal medium (LB, LB agar, M63 0.4% glucose). For each of the 3 types of medium, one sample was established as a reference (‘control’). The control samples were derived from cells grown in a respective medium without any additives and at 28 °C. When compared to the control samples, expression of *aiiO* was not altered above 1.5-fold in any of the tested conditions (Fig. 4). The >1-fold threshold was breached for LB culture in acidic pH 5.5, with respect to non-buffered LB medium of initial pH 7 (downregulation, −1.090 ± 0,116, p < 0.0005). The 1-fold threshold was also exceeded for LB agar cultures grown at 37 °C (upregulation, 1.320 ± 0.228) and at 28 °C in the presence of SCC3193 (1.286 ± 0.159), with respect to cultures grown separately at 28 °C, and in the middle exponential phase cells in LB with respect to early stationary phase cells in the same medium (1.023 ± 0,147). However, the latter three changes were not statistically significant (p > 0.05). Difference between M63% glucose and M63% glucose supplemented with 3OC12-HSL, although significant from a statistical standpoint, was below the 1-fold change cut-off (−0.115 ± 0.026, p < 0.05) (Fig. 4, Supplementary Table S4). In general, the expression of *aiiO* was not considerably induced under any of the conditions tested. In contrast, the expression of *groEL*, one of the least stable RG candidates in this study, was nearly 6-fold higher in samples from the mid-exponential culture in LB than in samples from LB agar at 37 °C, again with no significant induction observed for *aiiO* (Supplementary Fig. S3).

## Discussion

The QQ activity of *O. quorumnocens* A44 is attributed to the α/β hydrolase protein AiiO^32^. However, little is known concerning the role of this enzyme in the metabolism and environmental fitness of this strain.

Here, we developed a RT-qPCR assay to study the expression of *aiiO* gene in *O. quorumnocens* A44. A key consideration in RT-qPCR is the choice of stably expressed reference genes (RGs), also known as internal controls. To identify RGs suitable for RT-qPCR analyses in *O. quorumnocens* A44, we tested the expression stability of 11 candidate genes, 9 of which were previously reported as RGs in other bacterial species (i.a.^1–7^). Despite it is known that RGs suitable for one microorganism often do not show sufficient stability in others (RGs are not universal)^9^, and that the incorporation of inadequate genes to the normalization factor (NF) can significantly alter the results^12^, the experimental validation of the stability of RGs tends to be neglected in gene expression studies in prokaryotes.

Ideally, the expression stability of a potential RG should be validated in all physiological or experimental conditions intended for the study of the gene of interest^13^. Here, we ranked the stability of expression of 11 candidate RGs in a pilot assay including 10 different conditions, on samples originating from a single experiment, and then verified the performance of best RGs in the final dataset (11 culture conditions, all biological replicates). The strategy for applying a pilot assay to choose the most promising RG candidates is in line with practical recommendations for the implementations of MIQE^49^. When human clinical samples are analyzed, a minimum of 10 samples from different patients can be used to rank RG candidates and then the best scoring RGs, usually 2–5, are targeted alongside the gene(s) of interest for all samples/patients under investigation^50^. Although a sample set derived from a bacterium cultured in 10 different conditions is not equivalent to a sample set from 10 human individuals, the tools developed for the evaluation of gene expression stability usually require an input of this approximate size. Therefore, this approach is also used for prokaryotic studies^10^.

The expression stability of the candidate RGs was evaluated using geNorm^46^ and two other popular algorithms, NormFinder^47^ and BestKeeper^48^, each relying on a different statistical approach^9,51^. According to geNorm, the most stable RG candidates were *rho, gyrB* and *rpoD*. These genes were also among the five top ranking genes according to NormFinder. The ranking of gene stability suggested by Bestkeeper was different from that suggested by the other two algorithms. This method also yielded the smallest resolution, assigning good performance to the majority of tested genes. Interestingly, *recA*, listed among the top 4 genes by geNorm and 3 top genes by NormFinder, was among two with the worst ranks according to Bestkeeper. A discrepancy between the results from BestKeeper and the results from both geNorm and NormFinder were reported in other studies (i.e.^51,52^). The reason for that is different input data (raw C_q_ values for BestKeeper and relative expression data for geNorm and NormFinder) and different stability criteria considered by the three programs – a matter thoroughly summarized in the works of De Spiegelaere *et al*.^53^ and Gomes *et al*.^13^.

Based on the juxtaposition of the results from geNorm, NormFinder and BestKeeper, we chose *rho, rpoD* and *gyrB* as the most promising RG candidates to be used for the normalization of RT-qPCR data in *O. quorumnocens* A44. Importantly, these genes do not belong to a common functional group (*rho* – encoding the transcription termination factor Rho, *rpoD –* encoding the primary sigma factor σ^1^, *gyrB –* encoding the B subunit of DNA gyrase^1^) what reduces the chances that they are co-regulated^54,55^.

Apart from ranking the potential RGs based on their stability, the geNorm tool also suggests the optimal number of genes to be included to the normalization factor^46^. In this study, based on the pilot assay, geNorm suggested including three RGs in gene expression studies in A44. This value, however, is a guideline and does not have to be treated as a strict threshold^13,46^. We investigated whether and to what extent the number of genes in the NF influences our expression data. The results showed moderate impact of switching form 2-gene (*rho, gyrB*) to 3-gene (*rho, gyrB, rpoD*) NF, with a difference observed only for a single out of 11 investigated culture conditions. However, application of a single-gene NF (*rho*) significantly influenced the outcome under 3/11 and 4/11 culture conditions when compared to 2-gen and 3-gene NFs, respectively. These results are in line with the MIQE guidelines, according to which expression of the target gene(s) should be normalized to more than one RG^11^.

The expression stability of *rho, rpoD* and *gyrB*, the most promising RG candidates selected in the pilot assay, was validated on the final dataset (11 culture conditions, all biological replicates). The geNorm M and CV values obtained for these genes were, respectively, M = 0.441 and CV = 0.187 for *rho*, M = 0.453 and CV = 0.185 for *gyrB*, and M = 0.5 and CV = 0.221 in case of *rpoD*. According to Hellemans and coworkers^56^, the recommended threshold stability values depend on the type of analyzed samples. For homogeneous samples, defined in medical studies as such originating from cell cultures of the same cell type, these requirements are more stringent and amount to M < 0.5 and CV < 0.25. For heterogeneous samples, in medical studies defined as originating from different cell types, clinical biopsies and cancer tissue in general, the criteria are less stringent (M < 1 and CV < 0.5). Transposition of these thresholds to prokaryotic studies is not straightforward. However, recent publications concerning RT-qPCR in prokaryotes suggest that for a given bacterial strain cultured in different conditions, genes with geNorm values M < 1 can be applied for normalization of RT-qPCR data^10,13^. In this study, *rho, gyrB, rpoD* met both aforementioned thresholds for acceptable stability.

In strain A44, the 16S rRNA-coding gene, a popular RG candidate in bacteria^9^, has shown poor expression stability and was not considered as a component of NF. In some studies, however, the target may meet the stability criteria (see Gomes *et al*., 2018^13^). In such cases it should be considered that factors other than expression stability may also influence the performance of an RG. It was reported that while the turn-over of messenger RNAs in the cells is rather rapid, the ribosomal RNA is more stable and becomes degraded only under certain stress conditions^57^. These disproportions make a quantitative comparison between the two RNA types more difficult^13^. Another factor that comes to mind is the high disproportion in the C_q_ range observed between the potential genes of interest and the rRNA species, resulting from the high abundance of the latter in the cells. In RT-qPCR, however, as oppose to Northern blotting, it is not crucial for a good reference gene to be expressed at the same level as the gene of interest (Barbara D’haene, The gene expression blog, qbase+; https://blog.qbaseplus.com/four-tips-for-rt-qpcr-data-normalization-using-reference-genes).

A *gfp*-tagged derivative of the A44 strain^44^ was used throughout this study. Thus, apart from the genome-encoded RG candidates, we also investigated the stability of the heterologously expressed *gfp*. The gene is unique in terms of its occurrence in the terrestrial environment, therefore its stable expression in A44 GFP would create a low-cost alternative to the probe-based qPCR (i.e. TaqMan) for specific amplification of this RG candidate in complex samples. However, the stability of *gfp* in the tested setup proved insufficient. A potential cause may be uneven copy number per cell of the pPROBE-GTkan plasmid expressing *gfp*.

In this work, as a part of a broader study on the role of AiiO in the metabolism and environmental fitness of A44, we used RT-qPCR with the newly-established reference genes to investigate whether the expression of *aiiO* in A44 is altered upon exposure to AHLs and under a set of culture conditions. It is known that the expression of some enzyme-encoding genes may become highly elevated in the presence of cognate substrate(s), providing a solid link between a gene (protein) and a given compound or process. For example, Hommais *et al*.^58^ showed that the expression of *pelD*, a pectate lyase, can be induced over 10-fold in the stationary phase when polygalacturonate (substrate) is present.

The change in the expression of *aiiO* was investigated ninety minutes following the addition of synthetic AHLs (C6-HSL or 3OC12-HSL). The particular time point was chosen based on our previous experiments where the rate of exhaustion of AHLs following addition to cell culture was examined^32^. The consideration was to adjust the initial concentration of AHLs and time of sampling to prevent the total exhaustion of AHLs due to the activity of AiiO prior cell harvest.

Depending on the study, a gene is considered differentially regulated between two conditions when the change exceeds a 1-fold^59,60^ 1.5-fold^61,62^ or a 2-fold threshold^55^. Using RT-qPCR, we showed that the expression of *aiiO* in *O. quorumnocens* A44 is not upregulated (fold change <1^59,60^) upon supplementation with two synthetic AHLs, C6-HSL or 3OC12-HSL, nor in the presence of 3OC8-HSL and 3OC6-HSL and other compounds secreted to the medium by *P. parmentieri* SCC3193. No induction after addition of AHLs was reported earlier for *aiiB* and *blcC* genes encoding two QQ lactonases from *A. tumefaciens*^42,63^. Instead of being induced by signal molecules, the expression of those genes was enhanced in the presence of specific plant-derived compounds: succinic semialdehyde, gamma-hydroxybutyrate, gamma-butyrolactone, gamma-aminobutyrate and salicylic acid in case of *blcC*, and agrocinopine-enriched plant extracts in case of *aiiB*^42,63,64^. In this study, the expression of *aiiO* was investigated in the presence of water extract from potato roots. However, no significant up- or down-regulation of *aiiO* was observed in the applied setup.

Although the expression of *aiiO* was not distinctly upregulated in any of the conditions included in this study, we cannot exclude that such conditions exist. Some genes may be conserved in bacterial genomes, alike the *aiiO* homologues seem to be present in among different *Ochrobactrum* spp.^32,65^, even though they are useful only in a very particular niche or in the presence of certain environmental stress^66^. *Ochrobactrum* spp. are closely related to *Brucella* spp.^14^, known for their pathogenicity in a range of mammalian hosts^67^, and to *Agrobacterium* spp. and *Rhizobium* spp.^15^, known for establishing close interactions with plants^68^. Members of the *Ochrobactrum* genera were found in versatile environments, including soil, plants and the body of soil-dwelling organisms like the nematode *Caenorhabditis elegans* and the larvae of *Holotrichia parallela*^69^. Some *Ochrobactrum* spp. can cause opportunistic infections in humans, and one strain was proven to cause disease on mushrooms^70^. It is possible that the expression of *aiiO* becomes curtail (and therefore upregulated) in a very specific niche/host, upon certain abiotic stresses, or in the presence of some particular xenobiotic. Functional and structural similarities between the QQ enzymes and the xenobiotic/antibiotic-degrading enzymes were already denoted in several studies^71,72^.

In summary, we recommend *rho, gyrB* and *rpoD* as suitable RGs for gene expression analyses in *O. quorumnocens* A44. To our knowledge, this is the first study aimed to identify RGs for RT-qPCR analyses in a member of the *Ochrobactrum* genus. The expression of *aiiO*, a gene encoding the quorum quenching enzyme AiiO, was found not to be distinctly induced by the presence of AHLs, thereby providing no obvious indication that the primary substrate of AiiO are the AHLs.

## Materials and Methods

### Strains, chemicals and culture conditions

GFP-tagged derivative of *O. quorumnocens* A44 (A44 GFP)^44^, carrying plasmid pPROBE-GTkan^45^, was used throughout the experiments. The pVS1/p15a *ori* present on pPROBE-GTkan enables its stable propagation without selection^73,74^. The strain was grown under culture conditions listed in Table 1. Three types of basal media were used: Miller’s LB (Novagen, Germany), Miller’s LB agar (Novagen, Germany), and M63 mineral medium^75^. For the growth at pH 5.5, LB medium was buffered with 0.05M potassium hydrogen phthalate and the pH was adjusted to 5.5 with NaOH. To expose A44 GFP to metabolites secreted by the AHL-producing *P. parmentieri* SCC3931^76^, the two strains were co-cultured in form of two parallel lines (approx. 4 mm wide), streaked on LB agar next to each other (~2 mm distance). The co-culture approach was adopted from plate experiments designed to screen bacterial strains for the production of AHLs using color-developing biosensor strains^77^. A44 GFP was also exposed to two synthetic AHLs: *N*-hexanoyl-L-homoserine lactone (C6-HSL) (Fluka/Sigma-Aldrich, USA) and *N*-3-oxo-dodecanoyl-L-homoserine lactone (3OC12-HSL) (Quorum Sensing Nottingham, UK). Two mL of an overnight culture of A44 GFP in M63 with 0.4% glucose was supplemented with 50 μM·mL^−1^ of one of the signal molecules and incubated for 90 minutes prior to cell harvest. The potato root extract was obtained from the roots of 3-week-old potato plants (cv. Dalia) grown in sandy soil under growth chamber conditions: 22 ± 1 °C temperature, 85 ± 5% relative humidity, 16/8 h light/dark photoperiod. The roots were harvested, washed with distilled water and homogenized in a filter bag (Bioreba AG, Switzerland) after addition of sterile water in 1:1 (w/v) ratio. The resulting 50% water extract was centrifuged (21000 RCF, 10′) and the supernatant was filter-sterilized (0.2 µm, cellulose acetate). For bacterial culture, the extract was mixed 1:1 with 2× concentrated M63 medium and supplemented with glucose to the final concentration of 0.4%. The experiments involving synthetic AHLs and potato root extract were performed in a defined mineral medium with a single carbon source to minimize the potential influence of compounds present in LB. To assess the expression of *aiiO*, a minimum of two biological replicates were carried out for each tested culture condition (Supplementary Table S3). The number of samples representing each condition is not even. No samples additionally processed for technical trials were arbitrary excluded from the dataset, resulting with uneven sample size between the analyzed treatments.

### Selection of candidate RGs and primer design

The pool of candidate reference targets comprised 11 bacterial genes. Nine of them (16S rRNA, 23S rRNA, *dnaK*, *groEL*, *gyrB*, *recA*, *rho*, *rpoB*, *rpoD*) were chosen based on literature data. Homologues of these sequences were derived from the complete genome of *O. quorumnocens* A44 (CP022602.1–CP022605.1) (detailed list of loci in Table 2). The two other genes were: *gfp*, carried on the artificially introduced vector pPROBE-GTkan^45^ and CES85_4722, a hypothetical A44 gene with no data concerning its stability (a blind control). All PCR primers (Supplementary Table S1) were designed using the Primer3Plus software^78,79^ (http://primer3plus.com/). Specificity of the primers was verified using real-time PCR followed by melt curve analysis and electrophoresis in 1.2% agarose gel (TopVision, Thermo Scientific).

### Isolation of RNA and cDNA synthesis

Total RNA from bacterial monocultures was isolated using RNeasy Protect Bacteria Mini Kit according to the manufacturer’s protocol (Qiagen, Gemany), with the optional on-column DNAse digestion. Approximately 4.5 × 10^8^ cells were harvested and used per single isolation (cells harvested from 1 mL of cell suspension of turbidity equal to 5 units in McFarland’s scale). For cultures in liquid media, the turbidity was measured prior sampling and the volume of harvested cells was adjusted proportionally. Cells form solid media were suspended in sterile saline prior the turbidity measurement. The harvested cells (8000 RCF, 5′) were suspended in 500 μL of sterile saline to which 1 mL of RNA Protect Bacteria reagent (Qiagen, Gemany) was immediately added to prevent RNA decay.

All RNA samples were treated with TURBO DNA-free Kit (Thermo Scientific, USA) to remove any gDNA contamination and stored at −80 °C. The concentration and the quality of RNA for gene expression stability assessment was evaluated using an Agilent 2100 Bioanalyzer Instrument and an Agilent RNA 6000 Nano Kit (Agilent Technologies, USA). All processed samples had RNA Integrity value (RIN) ≥ 8.7. RIN values between 8–10 indicate intact, high quality RNA samples and RIN = 5.0 is the suggested minimum threshold for applicability of the given RNA in downstream RT-qPCR analyses^80,81^. Next, RNA was reverse-transcribed to cDNA with Transcriptor First Strand cDNA Synthesis Kit (Roche, Poland) using random hexamer primers. The optional denaturation step was included. Total amount of RNA used per reaction was 500 ng.

### qPCR

The assays were carried out using the CFX96 instrument (Bio-Rad), in a 96-well plate format. Reaction mixtures of 20 μL included: 2 × Power SYBR Green Mastermix (Thermo Scientific, USA), forward and reverse primers at a final concentration of 300 nM and 4 μL of diluted (1:3) post-RT mixture containing cDNA. The PCR conditions were 95 °C for 10 min, 40 cycles of 95 °C for 15S and 60 °C for 1 min, with a final melt curve 55–95 °C at a 0.5 °C/5S increment. Each reaction was performed in duplicate. The difference between duplicates was <0.5 cycle for 98% of reactions. Inter-run calibrator (IRC) was included in each subsequent plate. Parameters of the assay, including sensitivity, linearity and primer efficiency, were validated using a 6-point 10-fold serial dilution of templates, for which the corresponding post-PCR amplicons were applied.

### Expression stability and the optimal number of RGs

Eleven candidate RGs were ranked according to their expression stability under 10 culture conditions. Initially, the expression stability was evaluated in a geNorm^46^ pilot assay where a set of 10 cDNA samples (one per condition) was targeted for all RG candidates. geNorm incorporated into the qbase+ software, version 2.3 (Biogazelle NV, Belgium) was applied. In a geNorm pilot study, typically 8–10 candidate RGs are measured in a set of samples (usually 10). It is recommended that no sample type is overrepresented, what could introduce bias in terms of candidate genes being more stable in a given condition^55^. The resulting values (geNorm M) were compared with RG ranks obtained with NormFinder^47^ and BestKeeper^48^. Each of the applied methods is based on a different statistical approach, therefore the obtained results are not identical. To make our decision on the choice of a specific approach less arbitrary, we conducted a juxtaposition of the results obtained using the three algorithms by calculating a geometric mean. Genes with the best overall score were selected for further experiments. The expression stability the RGs pre-selected in the pilot was verified on the final dataset (11 culture conditions, 2–3 biological replicates) – the same one used to investigate the expression of *aiiO*. The validation was conducted using geNorm (M values).

The optimal number of RGs to be included in the normalization factor was established by calculating the V_n_/V_n + 1_ pair-wise variation, where n is the number of RGs in the NF^46^. In this method, a V value below 0.15 indicates no benefit of going from n to n + 1.

The performance of different normalization factors was compared on CNRQ values where the expression of *aiiO* was normalized to either one (NF1; *rho*), two (NF2; *rho, gyrB*) or three (NF3; *rho, gyrB, rpoD*) RGs. The statistical significance (α = 0.05) of pair-wise differences between groups normalized with different NFs was calculated using two-tailed Student’s t-test with unequal variation (Welch’s t-test).

### Analysis of gene expression

Gene expression analysis was conducted using the qbase+ software, version 2.3 (Biogazelle NV, Belgium). Subsequent steps were involved: the relative quantity (RQ) was calculated using ∆∆C_t_ method^82^, the RQ was normalized with respect to the established RGs (NRQ) and the value was calibrated with respect to the applied inter-run calibrator samples to obtain calibrated NRQ (CNRQ) interpreted as the relative expression. The significance of changes in the expression of *aiiO* between the ‘control’ and the ‘treated’ samples was evaluated using the BootstRatio tool for statistical analysis of fold-change (http://pdo.iconcologia.net/stats/br/index.html)^83^. To plot the fold change, CNRQ values were log base 2 transformed with respect to median of the respective reference samples for the three tested media types (LB agar, LB, M63 0.4% glucose).

## Supplementary information

Dataset 1

## Data Availability

All data generated or analysed during this study are included in this published article (and its Supplementary Information files).
