# Supplementary material for: Selection of reference genes for measuring the expression of aiiO in Ochrobactrum quorumnocens A44 using RT-qPCR
Source: Sci Rep. 2019 Sep 11;9:13129. doi: 10.1038/s41598-019-49474-6 (PMC6739375; doi:10.1038/s41598-019-49474-6)
Supplement: Supplementary file 1 — Dataset 1 [file 41598_2019_49474_MOESM1_ESM.docx]

**SUPPLEMENTARY DATA**

Selection of reference genes for measuring the expression of *aiiO* in *Ochrobactrum* *quorumnocens* A44 using RT-qPCR

Dorota M. Krzyżanowska ^a^, Anna Supernat ^b^, Tomasz Maciąg ^a^, Marta Matuszewska ^a^, Sylwia Jafra ^a,^ *

^a^ Laboratory of Biological Plant Protection, Intercollegiate Faculty of Biotechnology of University of Gdańsk and Medical University of Gdańsk, University of Gdańsk, ul. A. Abrahama 58, 80-307 Gdańsk, Poland;

^b^ Laboratory of Cell Biology, Intercollegiate Faculty of Biotechnology of University of Gdańsk and Medical University of Gdańsk, Medical University of Gdańsk, ul. Dębinki 1, 80-211 Gdańsk, Poland;

***** Correspondence: sylwia.jafra@biotech.ug.edu.pl; Tel.: +48-58-523-6315

**Supplementary Figures**

**Figure S1.** Specificity of the applied PCR primers**.** (A) Electrophoresis in 1.2% agarose gel showing single PCR products of expected sizes for each target gene. The negative control (NC) was prepared with primers targeting *aiiO*. GeneRuler 1 kb DNA Ladder (Thermo Scientific) was used for sizing of the DNA fragments. The arrows indicate fragment sizes in base pairs. Novel Juice (GeneDireX) was applied for DNA staining. The image was taken using ChemiDoc XSR (Bio-Rad) and processed using the Image Lab software. (B) Melt curve analysis (55-95 °C at a 0.5 °C / 5 s increment) for each target gene showing that the applied primers yield a single peak.


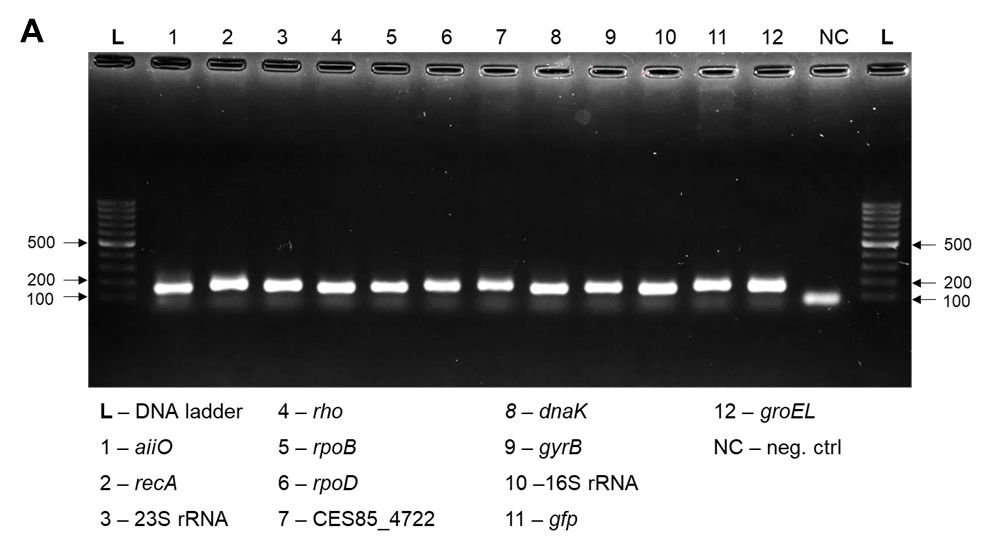


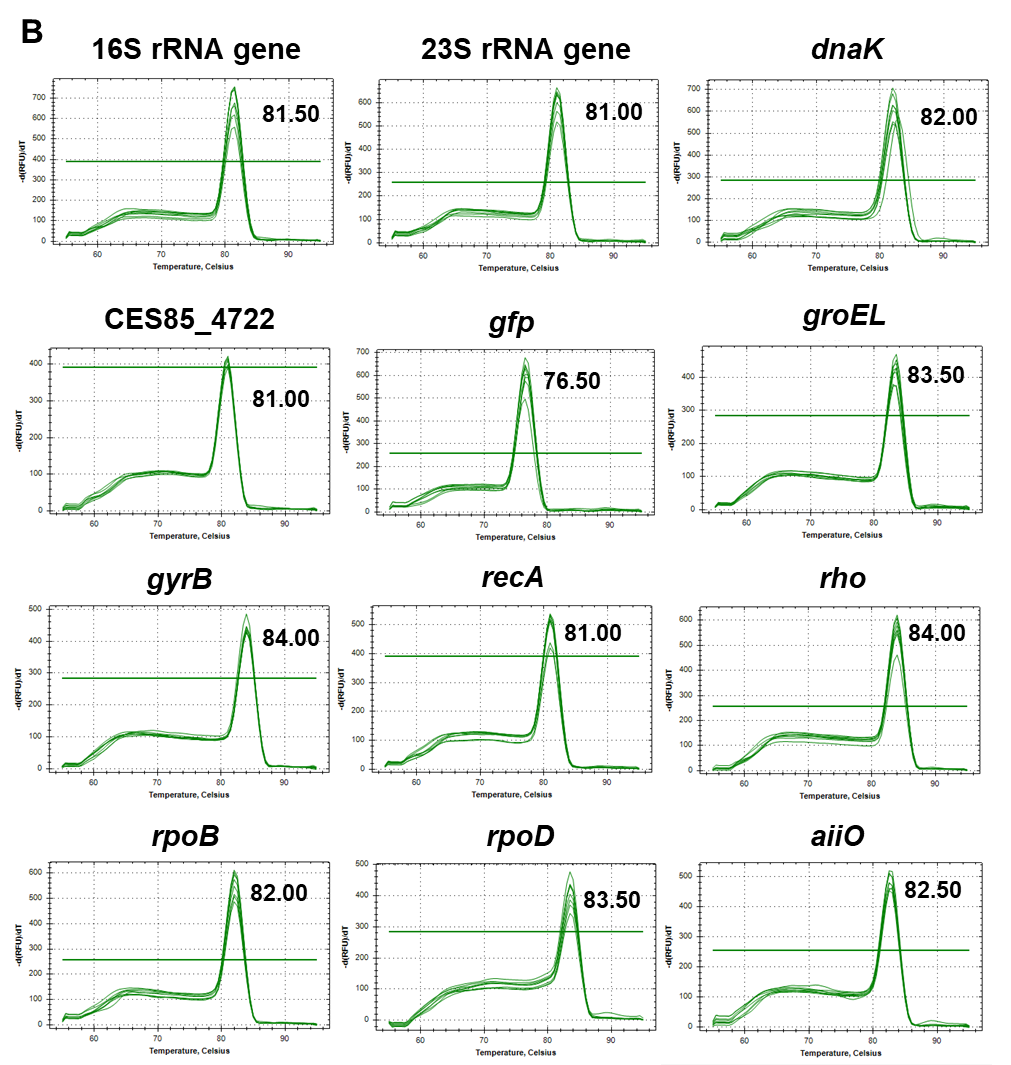


**Figure S2.** Standard curves for the estimation of PCR efficiency for *gyrB*, *rho, rpoD* (reference genes) and *aiiO* (studied gene). Numerical results are given in Table 2.


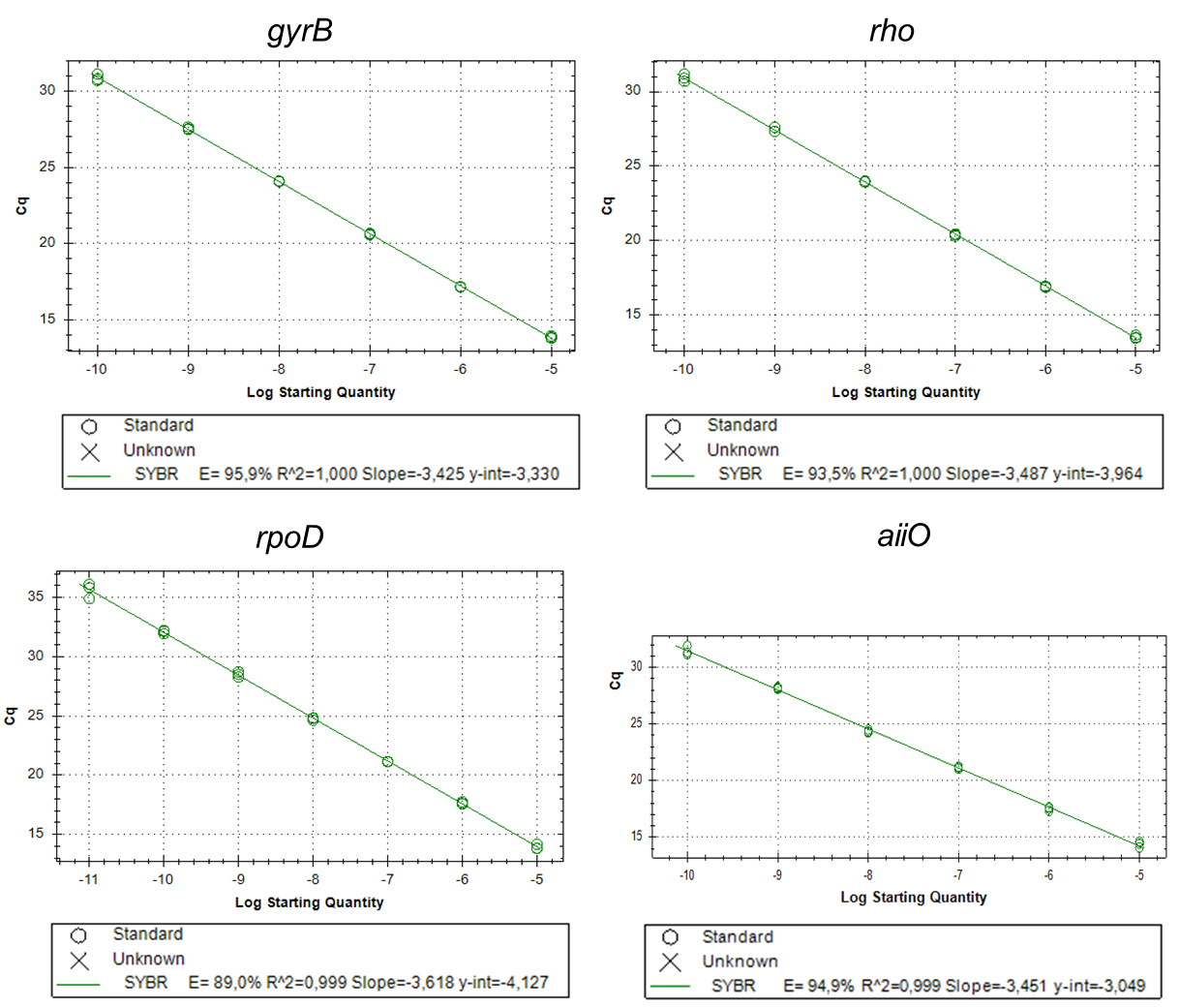


**Figure S3.** Significant upregulation of the expression of the *groEL* observed for A44 cells cultured LB at 28 °C during middle exponential growth phase when compared to cells from LB agar at 37 °C. In the same conditions, *aiiO* was not upregulated. The PCR efficiency of the *groEL* primers was 93%. The expression of both genes of interest was normalized to *rho*, *gyrB* and *rpoD* (NF3). Significance of change was calculated using BoostsRatio.


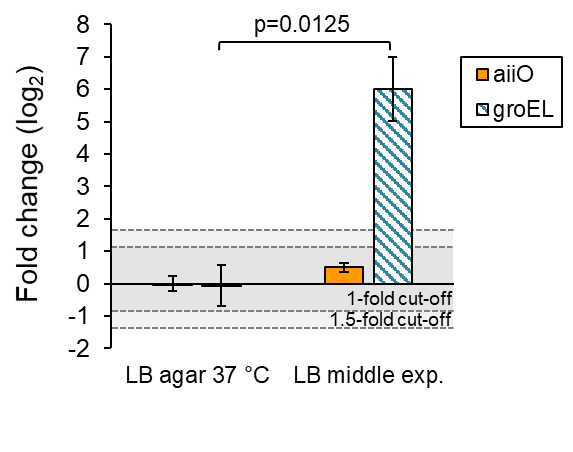


**Supplementary Tables**

**Table S1.** Primers designed and used in this study.

| **Target gene ^1^** | **Encoded protein/RNA** | **Primers** | **Primer sequences** | **Amplicon length (bp)** |
| --- | --- | --- | --- | --- |
| 16S rRNA | 16S rRNA component of the small subunit of the ribosome | F_16S rRNA_OP7  R_16S rRNA_OP7 | 5’ GCATGCTGATCCGCGATTAC  5’ ACACGTGCTACAATGGTGGT | 139 |
| 23S rRNA | 23S rRNA component of the large subunit of the ribosome | F_23S rRNA_OP7  R_23S rRNA_OP7 | 5’ ATTCGTGCAGGTCGGAACTT  5’ GTATAGGGTCTGACGCCTGC | 146 |
| *aiiO* | AiiO hydrolase inactivating the AHL-type signal molecules | F_aiiO_OP7  R_aiiO_OP7 | 5’ TGCGATTGATCCTGATCGCA  5’ AGCGCGCAATCATCTCGATA | 143 |
| *dnaK* | DnaK heat shock protein | F_dnaK_OP7  R_dnaK_OP7 | 5’ GTACCTGGTTGCCGAGTTCA  5’ GAACGGCAGGTTGATTTCGG | 142 |
| *gfp* | Green fluorescent protein (in this study constitutively expressed from vector pPROBE-GTkan) | F_gfp_OP7  R_gfp_OP7 | 5’ AGGGTGAAGGTGATGCAACA  5’ TGCCGTTTCATATGATCTGGGT | 148 |
| *groEL* | GroEL heat shock protein | F_groEL_OP7  R_groEL_OP7 | 5’ AGAAGGCTGAAATCGACGCA  5’ ATTTCTGTTGCACCGCCAAC | 148 |
| *gyrB* | DNA gyrase subunit B | F_ gyrB_OP7  R_ gyrB_OP7 | 5’ GTGAACGCGTTGTCGATCTG  5’ AAAGCTGACTTCGGTCCCAG | 144 |
| *recA* | Recombinase A | F_ recA_OP7  R_ recA_OP7 | 5’ TGCGCCTTGGTCAGAATGAT  5’ CGTCGTCTTACCCGAGCTTT | 146 |
| *rho* | Transcription termination factor Rho | F_ rho_OP7  R_ rho_OP7 | 5’ AAGGTTTTGACCGGTGGTGT  5’ GTCCATACGCGAACCGGTAT | 138 |
| *rpoB* | RpoB, the β subunit of bacterial RNA polymerase | F_ rpoB_OP7  R_ rpoB_OP7 | 5’ ACGCATGCCGATATCGTTCT  5’ GCCGTCTTGCGATAAACGTC | 143 |
| *rpoD* | RpoD (σ^70^), a “housekeeping” sigma factor involved in initiation of transcription in bacteria | F_ rpoD_OP7  R_ rpoD_OP7 | 5’ AAATGGGTTCCGTCGAGCTT  5’ TTCAGCTGTTCACGCCAGAT | 145 |
| CES85_4733 | hypothetical | F_ CES85_4733_OP7  R_ CES85_4733_OP7 | 5’ TCAGCGATGACATGCTCGAA  5’ CATGAATTCCTGCAGCAGCG | 140 |

^1^ Locus tags of the A44 genes in GenBank are as follows: 16S rRNA (CP022604.1: CES85_2017), 23S rRNA (CP022603.1: CES85_5624), *aiiO* (CP022604.1: CES85_2614), *dnaK* (CP022604.1: CES85_2678), *groEL* (CP022603.1: CES85_4825), *gyrB* (CP022604.1: CES85_0140), *recA* (CP022604.1: CES85_1344), *rho* (CP022604.1: CES85_2582), *rpoB* (CP022604.1: CES85_1384), *rpoD* (CP022604.1: CES85_1696), CES85_4733 (accordingly in CP022603.1). For *gfp*, the sequence was derived from the pPROBE-NT vector: AF286453.1, base pairs from 4066 to 4782.

Synthesis of oligonucleotides was outsourced to Sigma-Aldrich (USA).

**Table S2.** Expression stability of candidate RGs ranked using three popular algorithms and a geometric mean of the results.

| **geNorm** | | **NormFinder** | | **Bestkeeper** | | **Comprehensive^2^** | |
| --- | --- | --- | --- | --- | --- | --- | --- |
| **Gene** | **geNorm M** | **Gene** | **Stability value** | **Gene** | **r (p-value)** | **Gene** | **Geomean** |
| ***rho***^1^ | 0.528 | ***gyrB*** | 0.19 | *dnaK* | 0.001 | ***rho*** | 0.1046 |
| ***gyrB*** | 0.533 | *recA* | 0.28 | ***rho*** | 0.003 | ***rpoD*** | 0.1116 |
| ***rpoD*** | 0.570 | ***rpoD*** | 0.28 | 23S rRNA | 0.005 | ***gyrB*** | 0.1281 |
| *recA* | 0.613 | 16S rRNA | 0.72 | *groEL* | 0.005 | *dnaK* | 0.1285 |
| *rpoB* | 0.798 | ***rho*** | 0.79 | ***rpoD*** | 0.009 | 23S rRNA | 0.1676 |
| 16S rRNA | 0.913 | 23S rRNA | 1.00 | 16S rRNA | 0.009 | 16S rRNA | 0.1843 |
| 23S rRNA | 0.975 | *rpoB* | 1.36 | *rpoB* | 0.014 | *recA* | 0.2291 |
| *dnaK* | 1.020 | *dnaK* | 2.08 | ***gyrB*** | 0.021 | *rpoB* | 0.2493 |
| *gfp* | 1.058 | *gfp* | 3.32 | *recA* | 0.071 | *groEL* | 0.3285 |
| *groEL* | 1.153 | *groEL* | 6.05 | *gfp* | 0.261 | *gfp* | 0.9713 |
| CES85_4722 | 1.273 | CES85_4722 | 8.32 | CES85_4722 | - | CES85_4722 | - |

^1^ shown in bold are the genes selected for normalization of the expression of the target gene *aiiO*

^2^ juxtaposition of results obtained using geNorm, NormFinder and Bestkeeper based on the calculation of geometric mean (Geomean)

**Table S3.** Relative expression (CNRQ) of the *aiiO* gene with respect to NF3 (*rho,* *gyrB* and *rpoD*).

| **Sample** |  | **Biol. replicate** | **CNRQ** | **Median** | **SD** |
| --- | --- | --- | --- | --- | --- |
| LB agar, O/N^4^ | 28 °C | A | 1.189 | 1.341 | 0.166 |
|  |  | B | 1.341 |  |  |
|  |  | C | 1.520 |  |  |
|  | 20 °C | A | 2.781 | 2.456 | 0.459 |
|  |  | B | 2.131 |  |  |
|  | 37 °C | A | 2.994 | 3.369 | 0.530 |
|  |  | B | 3.743 |  |  |
|  | 28 °C, co-culture SCC3193 | A | 3.535 | 3.281 | 0.360 |
|  |  | B | 3.026 |  |  |
| LB | early stationary | A | 2.556 | 2.340 | 0.439 |
|  |  | B | 2.340 |  |  |
|  |  | C | 1.710 |  |  |
|  | middle exponential | A | 5.212 | 4.834 | 0.479 |
|  |  | B | 4.834 |  |  |
|  |  | C | 4.262 |  |  |
|  | early stationary, pH 5.5 | A | 1.163 | 1.100 | 0.089 |
|  |  | B | 1.038 |  |  |
| M63 | no supplementation | A | 2.656 | 2.983 | 0.462 |
| 0.4% glucose |  | B | 3.310 |  |  |
|  | C6-HSL | A | 3.288 | 2.684 | 0.853 |
|  |  | B | 2.081 |  |  |
|  | 3OOC12-HSL | A | 2.791 | 2.756 | 0.050 |
|  |  | B | 2.721 |  |  |
|  | root extract | A | 3.968 | 3.845 | 0.175 |
|  |  | B | 3.721 |  |  |

^1^ co-culture SCC3193 – growth in the presence of AHLs secreted by *P. parmentieri* SCC3193; C6-HSL, 3OC12-HSL –expression of *aiiO* 90 min after the addition of 50 μM·mL^-1^ of the respective AHL; root extract – growth in the presence of 25% water extract from potato roots.

^2^ CNRQ, relative expression with respect to reference genes *rho, gyrB and rpoD*

^3^ SD – standard deviation

^4^ O/N – overnight culture

**Table S4.** Results of the statistical analysis performed with BootstRatio to evaluate the significance of differences of fold-change in the expression of *aiiO* (normalized to NF3) with respect to 3 reference samples, one per each of the applied media types (A-C).

| 1. Reference sample (CTRL): **LB agar at 28 °C (LA28)** | | | | | | | | | | | | | | | p< | | | | |
| --- | --- | --- | --- | --- | --- | --- | --- | --- | --- | --- | --- | --- | --- | --- | --- | --- | --- | --- | --- |
| Type ^1^ | Mean.  Type | Median.  Type | SE.  Type | Mean.Ctrl | Median.Ctrl | SE.  Ctrl | Ratio.  Mean.  Obs | N.  Type | N.  Ctrl | Mean.  Ratio | Median.  Ratio | SD.  Ratio | Prob.  Ratio>1 | Prob.  Ratio<1 | 0.1 | 0.05 | 0.01 | 0.001 | 0.0005 |
| **LA20 ^2^** | **2.456** | **2.456** | **0.325** | **1.350** | **1.341** | **0.096** | **1.819** | **2** | **3** | **1.137** | **1.135** | **0.650** | **0.557** | **0.442** | **N** | **N** | **N** | **N** | **N** |
| **LA3193** | **3.281** | **3.281** | **0.255** | **1.350** | **1.341** | **0.096** | **2.430** | **2** | **3** | **1.450** | **1.441** | **0.844** | **0.659** | **0.341** | **N** | **N** | **N** | **N** | **N** |
| **LA37** | **3.369** | **3.369** | **0.375** | **1.350** | **1.341** | **0.096** | **2.495** | **2** | **3** | **1.494** | **1.471** | **0.889** | **0.671** | **0.328** | **N** | **N** | **N** | **N** | **N** |
| LB ESTAT | 2.202 | 2.340 | 0.254 | 1.350 | 1.341 | 0.096 | 1.631 | 3 | 3 | 1.087 | 1.057 | 0.569 | 0.537 | 0.463 | N | N | N | N | N |
| LB MEXP | 4.769 | 4.834 | 0.276 | 1.350 | 1.341 | 0.096 | 3.533 | 3 | 3 | 2.227 | 2.150 | 1.145 | 0.836 | 0.164 | N | N | N | N | N |
| LB pH5 | 1.101 | 1.101 | 0.063 | 1.350 | 1.341 | 0.096 | 0.815 | 2 | 3 | 0.478 | 0.479 | 0.284 | 0.000 | 1.000 | Y**^3^** | Y | Y | Y | Y |
| M63 | 2.983 | 2.983 | 0.327 | 1.350 | 1.341 | 0.096 | 2.210 | 2 | 3 | 1.347 | 1.316 | 0.773 | 0.635 | 0.364 | N | N | N | N | N |
| M63 OC12 | 2.756 | 2.756 | 0.035 | 1.350 | 1.341 | 0.096 | 2.042 | 2 | 3 | 1.143 | 1.142 | 0.668 | 0.569 | 0.431 | N | N | N | N | N |
| M63C6 | 2.685 | 2.685 | 0.604 | 1.350 | 1.341 | 0.096 | 1.989 | 2 | 3 | 1.308 | 1.329 | 0.792 | 0.624 | 0.376 | N | N | N | N | N |
| M63ROOT | 3.845 | 3.845 | 0.124 | 1.350 | 1.341 | 0.096 | 2.848 | 2 | 3 | 1.687 | 1.724 | 0.949 | 0.725 | 0.275 | N | N | N | N | N |
|  |  |  |  |  |  |  |  |  |  |  |  |  |  |  |  |  |  |  |  |
| 1. Reference sample (CTRL): **LB early stationary (LB ESTAT)** | | | | | | | | | | | | | | | p< | | | | |
| Type | Mean.  Type | Median.  Type | SE.  Type | Mean.Ctrl | Median.Ctrl | SE.  Ctrl | Ratio.  Mean.  Obs | N.  Type | N.  Ctrl | Mean.  Ratio | Median.  Ratio | SD.  Ratio | Prob.  Ratio>1 | Prob.  Ratio<1 | 0.1 | 0.05 | 0.01 | 0.001 | 0.0005 |
| **LB MEXP** | **4.769** | **4.834** | **0.276** | **2.202** | **2.340** | **0.254** | **2.166** | **3** | **3** | **1.597** | **1.622** | **0.806** | **0.733** | **0.267** | **N** | **N** | **N** | **N** | **N** |
| **LB pH5** | **1.101** | **1.101** | **0.063** | **2.202** | **2.340** | **0.254** | **0.500** | **2** | **3** | **0.330** | **0.334** | **0.195** | **0.000** | **1.000** | **Y** | **Y** | **Y** | **Y** | **Y** |
| LA28 | 1.350 | 1.341 | 0.096 | 2.202 | 2.340 | 0.254 | 0.613 | 3 | 3 | 0.437 | 0.432 | 0.245 | 0.000 | 1.000 | Y | Y | Y | Y | Y |
| LA20 | 2.456 | 2.456 | 0.325 | 2.202 | 2.340 | 0.254 | 1.115 | 2 | 3 | 0.762 | 0.746 | 0.440 | 0.321 | 0.679 | N | N | N | N | N |
| LA3193 | 3.281 | 3.281 | 0.255 | 2.202 | 2.340 | 0.254 | 1.490 | 2 | 3 | 1.005 | 1.003 | 0.587 | 0.504 | 0.495 | N | N | N | N | N |
| LA37 | 3.369 | 3.369 | 0.375 | 2.202 | 2.340 | 0.254 | 1.530 | 2 | 3 | 1.065 | 1.037 | 0.604 | 0.524 | 0.476 | N | N | N | N | N |
| M63 | 2.983 | 2.983 | 0.327 | 2.202 | 2.340 | 0.254 | 1.355 | 2 | 3 | 0.881 | 0.849 | 0.536 | 0.404 | 0.596 | N | N | N | N | N |
| M63 OC12 | 2.756 | 2.756 | 0.035 | 2.202 | 2.340 | 0.254 | 1.252 | 2 | 3 | 0.783 | 0.791 | 0.462 | 0.352 | 0.648 | N | N | N | N | N |
| M63 C6 | 2.685 | 2.685 | 0.604 | 2.202 | 2.340 | 0.254 | 1.219 | 2 | 3 | 0.883 | 0.860 | 0.547 | 0.407 | 0.593 | N | N | N | N | N |
| M63ROOT | 3.845 | 3.845 | 0.124 | 2.202 | 2.340 | 0.254 | 1.746 | 2 | 3 | 1.131 | 1.140 | 0.668 | 0.563 | 0.438 | N | N | N | N | N |
|  |  |  |  |  |  |  |  |  |  |  |  |  |  |  |  |  |  |  |  |
| 1. Reference sample (CTRL): **M63 0.4% glucose (M63)** | | | | | | | | | | | | | |  | p< | | | | |
| Type | Mean.  Type | Median.  Type | SE.  Type | Mean.Ctrl | Median.Ctrl | SE.  Ctrl | Ratio.  Mean.  Obs | N.  Type | N.  Ctrl | Mean.  Ratio | Median.  Ratio | SD.  Ratio | Prob.  Ratio>1 | Prob.  Ratio<1 | 0.1 | 0.05 | 0.01 | 0.001 | 0.0005 |
| **M63 C6** | **2.685** | **2.685** | **0.604** | **2.983** | **2.983** | **0.327** | **0.900** | **2** | **2** | **0.596** | **0.592** | **0.353** | **0.163** | **0.837** | **N** | **N** | **N** | **N** | **N** |
| **M63 OC12** | **2.756** | **2.756** | **0.035** | **2.983** | **2.983** | **0.327** | **0.924** | **2** | **2** | **0.507** | **0.519** | **0.305** | **0.043** | **0.956** | **Y** | **Y** | **N** | **N** | **N** |
| **M63ROOT** | **3.845** | **3.845** | **0.124** | **2.983** | **2.983** | **0.327** | **1.289** | **2** | **2** | **0.734** | **0.737** | **0.422** | **0.301** | **0.699** | **N** | **N** | **N** | **N** | **N** |
| LB ESTAT | 2.202 | 2.340 | 0.254 | 2.983 | 2.983 | 0.327 | 0.738 | 3 | 2 | 0.485 | 0.472 | 0.257 | 0.000 | 1.000 | Y | Y | Y | Y | Y |
| LB MEXP | 4.769 | 4.834 | 0.276 | 2.983 | 2.983 | 0.327 | 1.599 | 3 | 2 | 1.030 | 1.058 | 0.533 | 0.531 | 0.470 | N | N | N | N | N |
| LB pH5 | 1.101 | 1.101 | 0.063 | 2.983 | 2.983 | 0.327 | 0.369 | 2 | 2 | 0.210 | 0.213 | 0.126 | 0.000 | 1.000 | Y | Y | Y | Y | Y |
| LA28 | 1.350 | 1.341 | 0.096 | 2.983 | 2.983 | 0.327 | 0.453 | 3 | 2 | 0.304 | 0.313 | 0.154 | 0.000 | 1.000 | Y | Y | Y | Y | Y |
| LA20 | 2.456 | 2.456 | 0.325 | 2.983 | 2.983 | 0.327 | 0.823 | 2 | 2 | 0.492 | 0.482 | 0.299 | 0.036 | 0.962 | Y | Y | N | N | N |
| LA3193 | 3.281 | 3.281 | 0.255 | 2.983 | 2.983 | 0.327 | 1.100 | 2 | 2 | 0.645 | 0.627 | 0.369 | 0.218 | 0.781 | N | N | N | N | N |
| LA37 | 3.369 | 3.369 | 0.375 | 2.983 | 2.983 | 0.327 | 1.129 | 2 | 2 | 0.707 | 0.711 | 0.397 | 0.276 | 0.725 | N | N | N | N | N |

^1^ “Type” refers to given conditions the A44 cells were cultured in. The abbreviations applied in the table can be decoded by referring to Table 1, summarizing all the culture conditions applied in this study. Shown in bold at the top of each section of the Table (A-C) are the samples derived from bacterial cultures in the same basal medium as the reference sample.

^2^ samples from the same medium type as the reference (CTRL) were shown in bold

^3^ where the statistical significance at the declared level was reached (Y), the cells were highlighted grey. N stands for no statistical significance at the declared level.
